# Facing the unforeseen: coping, meaning in life, fear of death, and their associations across the COVID-19 pandemic and the early phase of Russia’s full-scale invasion of Ukraine—a repeated cross-sectional study in independent Hungarian samples

**DOI:** 10.3389/fpsyt.2026.1916005

**Published:** 2026-10-28

**Authors:** Petra Szimon, Nandor Takacs, Agnes Zana

**Affiliations:** 1 Semmelweis University, Faculty of Medicine, Institute of Behavioral Sciences, Budapest, Hungary; 2 Károli Gáspár University of the Reformed Church in Hungary, Faculty of Humanities and Social Sciences, Institute of Psychology, Department of Personality- and Health Psychology, Budapest, Hungary; 3 Independent Researcher, Budapest, Hungary

**Keywords:** coping, COVID-19, fear of death, meaning in life, mental health, Russia’s full-scale invasion of Ukraine

## Abstract

**Background:**

The coronavirus disease 2019 (COVID-19) pandemic and Russia’s full-scale invasion of Ukraine represent unprecedented global stressors with substantial consequences for mental health.

**Objective:**

A repeated cross-sectional study was conducted in three independent Hungarian samples before COVID-19 (*N* = 249), after the second wave of COVID-19 (*N* = 265), and during the early phase of Russia’s full-scale invasion of Ukraine (*N* = 148) to examine differences in meaning in life, coping competencies, and fear of death, as well as in the relationships among these constructs. The outbreak of the invasion during data collection provided a unique opportunity to examine these constructs during its early phase.

**Results:**

Compared with the post-COVID sample, lower descriptive levels of fear of death were observed following the outbreak of Russia’s full-scale invasion of Ukraine. Lower levels of several coping resources, particularly sense of coherence and social competencies, together with the greater relevance of search for meaning, were among the key findings associated with these unforeseen stressors. Stronger associations between coping and meaning in life were identified, while the association between presence of meaning and search for meaning became stronger following the outbreak of the invasion. Network analyses further suggested a closer integration of meaning-related processes with fear of death and coping resources, although these patterns should be interpreted cautiously given the limited stability of the network estimates.

**Conclusion:**

The findings suggest that exposure to successive unforeseen large-scale stressors may be associated with the reorganization of meaning-related processes and coping resources. The patterns observed following the outbreak of the war may be interpreted within the framework of distal defense processes described by the terror management theory, although these mechanisms were not directly assessed in the present study. The results suggest that longer-term existential anxiety processes and their delayed psychological and social consequences should be considered when developing mental health interventions. These findings may contribute to the refinement of healthcare interventions across multiple levels and cultural contexts in preparation for current and future crises.

## Introduction

Death anxiety is a common aspect of human existence, and the way individuals cope with it may influence quality of life and psychological wellbeing. Humans are the only species capable of consciously reflecting on their own mortality. This unique capacity may also represent a psychological burden (1, 2). The contemplation of death acts as a significant psychological stressor, primarily because of its uncontrollable and inevitable nature. It often triggers death anxiety, feelings of helplessness, loss of control, and a sense of meaninglessness (3).

The coronavirus disease 2019 (COVID-19) pandemic and Russia’s full-scale invasion of Ukraine are both considered uncontrollable stressors that expose populations to high levels of uncertainty and have been associated with adverse mental health outcomes. Several studies have explored the detrimental impacts of these crises and the ways individuals have responded to them (4–11). Recent research suggests that the pandemic exerts a more persistent and direct impact on mental health than armed conflicts (12, 13). Higher anxiety levels associated with war-related threats have also been reported (4). It has also been suggested that, beyond the profound effects of the COVID-19 pandemic, the combined impact of both crises should be considered (4, 14). In the present study, the outbreak of Russia’s full-scale invasion of Ukraine is conceptualized as an unforeseen existential boundary situation rather than a prolonged war-related exposure.

The terror management theory (TMT) and the existential paradigm view large-scale death-related events—such as pandemics and war-related crises—as mortality salience triggers. Such events may intensify death-related concerns and contribute to elevated psychological distress.

Both early existential writings and subsequent TMT research suggest that encounters with mortality evoke emotional discomfort, anxiety, and defensive coping responses (15–19). At the proximal level, thoughts of death may elicit defensive responses such as denial and avoidance (20). According to TMT, self-esteem and culturally shared worldviews function as distal anxiety-buffering mechanisms that help individuals manage mortality-related concerns (20–24). Rogers, Maslow, and Erikson suggested additional personal characteristics that contribute to managing the threat of death: self-actualization, openness to experience, sense of integrity, and a positive and coherent self-perception (19). Recent studies have revisited coping strategies and principles that may help individuals adapt to severe circumstances, such as pandemic- and war-related crises (25–28). Self-efficacy, social support, optimism, and problem-focused coping have consistently emerged as some of the most important protective resources identified in these studies.

Ineffective or maladaptive coping may contribute to the development of mental disorders (e.g., psychosomatic disorders, anxiety disorders, or depression) (24). Research has shown that death is one of the most common worry themes underlying generalized anxiety disorder (GAD), compromising an individual’s quality of life and psychosocial functioning (24, 29, 30). Similar adverse mental health outcomes have also been associated with pandemic- and war-related trauma, including direct and indirect exposure to life-threatening situations and death-related stressors (7, 10–14, 25–28, 31–36).

Experimental research indicates that, in response to mortality salience, people attempt to sustain the belief that human life is meaningful (23, 37). Several studies have examined the relationship between meaning in life (MIL) and death anxiety. Routledge and Juhl (23) found that participants with a stronger sense of MIL experienced weaker death anxiety when they were exposed to a death prime. MIL has also been associated with greater emotional wellbeing (38–40) and more effective coping with stressful life events (41, 42). This perspective is further elaborated in Wong’s dual-system model of meaning management theory (MMT) (43), which states that an individual can cope with death anxiety and achieve a good life by striving for life-enhancing goals (44). Viktor E. Frankl (45, 46) emphasized the importance of MIL in his logotherapy as a key coping strategy, placing the search for meaning at its core, even under the most futureless circumstances. Building on these existential perspectives, contemporary conceptualizations distinguish between different dimensions of MIL. According to Steger’s model, MIL comprises two related but distinct dimensions: the presence of meaning, reflecting the perceived meaningfulness of one’s life, and the search for meaning, reflecting the motivation to find or deepen MIL (47, 48).

Besides MIL, personality characteristics and cognitive coping processes may provide adaptive psychological defense mechanisms at the individual level. Numerous studies over the past decades have identified coping processes and personality traits that contribute to psychological adaptation. Lazarus introduced a three-step appraisal process in which primary and secondary appraisal phases define how the individual responds to a stressor. Whether a stressor is appraised as a threat or a controllable challenge influences the coping process (49). Positive emotional adjustment as an early response facilitates psychological adaptation to uncontrollable circumstances (23).

Contemporary coping research has identified meaning-making (e.g., positive reinterpretation) and problem-focused coping, which involves active efforts to address stressors, as important coping dimensions (50). Ward et al. (50) explored how the cognitive, affective, and motivational aspects of MIL influence meaning-focused, problem-focused, and emotion-focused coping, emphasizing the role of adaptive coping strategies and coping self-efficacy. Their findings highlighted the complex relationships between MIL and coping. Halama (41) demonstrated that MIL moderates the relationship between perceived stress and coping and that a sense of meaning serves as a buffer against stress. Recent studies examining the consequences of Russia’s full-scale invasion of Ukraine for mental health have consistently reported elevated levels of stress, anxiety, depressive symptoms, and post-traumatic stress among affected populations (12, 51–53). A nationwide study by Lushchak et al. further demonstrated a substantial psychological burden among non-displaced persons, internally displaced persons, and refugees across Ukraine (53). Using network analytic approaches, previous studies have further identified anxiety as a central variable linking stress, depression, and reduced life satisfaction (12). Similarly, Chudzicka-Czupała et al. (11) highlighted that the closer a country is to the war-affected area, the greater the psychological impact of media coverage as a source of information. Media exposure has become an important source of information regarding death-related, crisis-related, and war-related events and may shape attitudes toward mortality and perceived threat (54, 55). Exposure to trauma-related media content may adversely affect mental health and contribute to sustained stress and anxiety (13). Although these studies have substantially advanced our understanding of the psychological consequences of large-scale existential threats, considerably less is known about how the relationships between fear of death, MIL, and coping competencies differ across successive global crises in neighboring populations.

The present study was conducted in the Hungarian population, extending the focus beyond university students to include a broader adult population and thereby providing more comprehensive information relevant to mental health interventions. Using targeted snowball sampling, we recruited medical professionals, non-medical helping professionals, and non-helping professionals. This approach provided broader, although non-representative, insight into the psychological impacts of the pandemic and Russia’s full-scale invasion of Ukraine across different segments of society, rather than focusing exclusively on university students or healthcare workers, the populations most commonly examined in related research.

Hungary’s geographical proximity to Ukraine and its involvement in the resulting migration crisis represent distinctive contextual factors. In addition, media coverage has continuously emphasized the country’s exposure to this armed conflict. To date, within Europe, the psychological consequences associated with the COVID-19 pandemic and Russia’s full-scale invasion of Ukraine have primarily been investigated in Ukrainian, Polish, and East German populations, mostly several months after the outbreak of the war.

An armed conflict can be understood as an existential boundary situation, rendering the original research questions particularly relevant in the context of Russia’s full-scale invasion of Ukraine. The outbreak of the war provided an opportunity to examine psychological responses associated with an additional unforeseen existential stressor. The sample size allowed for the investigation of between-sample differences that may reflect psychological responses observed shortly after the outbreak of Russia’s full-scale invasion of Ukraine during a period marked by increased migration and heightened societal attention to the nearby armed conflict. These findings may contribute to understanding the psychological implications of such crises at both personal and collective levels.

Populations exposed to pandemic-related and geopolitical crises require preventive and supportive mental health programs for which healthcare systems need to be prepared. Citizens in this region have been exposed simultaneously to direct or indirect life-threatening challenges associated with both the pandemic and the nearby armed conflict. Hungary’s geographical proximity to the conflict may have increased perceived threat and psychological burden, similar to observations reported in other populations living closer to the war zone (11).

Post-traumatic symptoms have been observed regardless of the cultural context (11). The relevance of the present study lies in examining these processes in a Hungarian sample from an EU member state located in geographical proximity to the conflict.

The findings may contribute to a broader understanding of trends in mental health processes and to identifying potential future risks and intervention opportunities. Besides the investigated coping competencies, examining the relationship between fear of death and MIL can provide important insights for the design of appropriate intervention programs.

Based on TMT and previous research, we expected that the COVID-19 pandemic and the subsequent outbreak of Russia’s full-scale invasion of Ukraine would be associated with differences in fear of death, coping competencies, and MIL across the three study periods. We further expected that the associations among these constructs would differ across the three independent samples, potentially reflecting psychological adaptation to consecutive collective existential stressors.

Accordingly, the present study aimed to examine between-sample differences in fear of death, MIL, coping competencies, and their interrelationships across three independent Hungarian samples collected before the COVID-19 pandemic, after the second wave of the pandemic, and following the outbreak of Russia’s full-scale invasion of Ukraine.

## Materials and methods

### Study design

This repeated cross-sectional study included three independent Hungarian samples. The pre-COVID sample was collected between June and November 2019. The post-COVID data collection began on 11 January 2022, following the COVID-19 wave associated with the second-highest mortality in Hungary, and continued until 20 August 2022. Based on the date of questionnaire completion, participants in the post-COVID sample were further classified into those who responded before or after the outbreak of Russia’s full-scale invasion of Ukraine on 24 February 2022. This classification reflects the timing of data collection rather than individual exposure to war-related events. Because the samples were independent, the study design does not allow for within-person longitudinal comparisons.

### Participants and data collection

#### Eligibility criteria

Eligible participants were Hungarian adults who voluntarily completed the anonymous online questionnaire.

The study included 662 Hungarian participants recruited through targeted snowball sampling using an online questionnaire administered via Google Forms. The pre-COVID sample comprised 249 participants, whereas the post-COVID sample included 413 participants. Of the latter, 265 completed the survey before and 148 after the outbreak of Russia’s full-scale invasion of Ukraine. The classification of participants according to whether data were collected before or after the outbreak of Russia’s full-scale invasion of Ukraine reflects only the timing of data collection and does not capture individual exposure to war-related events or media coverage.

Participants were recruited through professional organizations and closed online communities, including the Napfogyatkozás Association Bereavement Counsellor Group, the Hungarian Death Café Community, the Mental Health Experts Community, and the Psychologist Community.

Participants were classified into three occupational groups: medical professionals, non-medical helping professionals, and non-helping professionals.

The post-COVID questionnaire was an expanded version of the previous questionnaire and included additional sociodemographic and psychological variables, including sex, age, profession, religiosity, region of residence, self-awareness, death awareness, self-care, and pandemic-related factors. Only sex, age, and profession were assessed consistently across all three study periods. Regional information was available only for the PostCoPostW sample. In addition, religiosity was assessed using different measures across the study periods, precluding direct comparisons across all three samples.

#### Instruments

The validated Hungarian versions of the questionnaires discussed below were employed in the study.

*Meaning in Life Questionnaire (MLQ)* (47, 48): The MLQ assesses two dimensions of MIL using 10 items rated on a seven-point scale from “absolutely true (7)” to “absolutely untrue (1).” The Presence of Meaning subscale (MLQ-P) assesses the extent to which respondents feel their lives are meaningful. The Search for Meaning subscale (MLQ-S) assesses the extent to which respondents are motivated to seek or deepen meaning in their lives (48). For the MLQ-P subscale, McDonald’s *ω* = 0.88 and Cronbach’s *α* = 0.85; for the MLQ-S subscale, McDonald’s *ω* = 0.85 and Cronbach’s *α* = 0.85.

*Psychological Immune System Inventory (PISI)* (56): The 80-item PISI assesses 16 coping potential competencies within three cognitive, dynamically interacting systems: Monitoring-Approach System (MAS); Mobilizing, Creating and Executing System (MCES); and Self-Regulatory System (SRS). The competencies are multidimensional, incorporating cognitive, behavioral, and trait-like factors, which serve as coping resources. Items are rated on a four-point Likert scale from “very true (4)” to “very untrue (1).” The ranges for internal consistency estimates were as follows: McDonald’s *ω* = 0.62–0.88 and Cronbach’s *α* = 0.61–0.88.

*The Multidimensional Fear of Death Scale (MFODS)* (57, 58): The MFODS consists of 42 items categorized into eight factors. The factors are as follows: fear of the dying process, fear of the dead, fear of being destroyed, fear for significant others, fear of the unknown, fear of conscious death, fear for the body after death, and fear of premature death.

Items are rated on a five-point Likert scale ranging from “strongly agree (1)” to “strongly disagree (5).” The original scale is reverse-scored; therefore, the items were recoded so that higher scores indicate greater fear of death (FOD) (57, 59–61). The ranges for internal consistency estimates were as follows: McDonald’s *ω* = 0.61–0.80 and Cronbach’s *α* = 0.58–0.80.

### Statistical analysis

Items with missing responses were excluded only from analyses involving the respective variables. Consequently, the valid sample size varied slightly across analyses.

Pearson’s correlation coefficients (*r*) were calculated to examine associations among the study variables. Fisher’s *Z* tests were conducted to compare selected correlation coefficients across the three independent samples.

Comparisons between the three independent samples were performed using one-way analyses of variance (ANOVA). When overall group differences were significant, Games–Howell *post hoc* tests were conducted because group sizes were unequal and the assumption of homogeneity of variances was not consistently met. Cohen’s *d* was calculated to estimate pairwise effect sizes. Between-sample differences in categorical sociodemographic variables were examined using Pearson’s chi-square tests. Effect sizes were estimated using Cramer’s *V*.

Backward linear regression analyses were performed separately for each study period as an exploratory, data-driven procedure to identify variables predicting FOD from the same predefined set of candidate predictors. Regression assumptions were assessed using residual-versus-predicted plots, Q–Q plots of standardized residuals, Cook’s distance, and collinearity diagnostics. No substantial violations of linearity or homoscedasticity were observed, residual distributions showed only minor deviations from normality, and Cook’s distance remained below 1 in all cases. Multicollinearity diagnostics were within acceptable ranges (VIF ≤ 1.9; tolerance ≥ 0.54).

Network structures were estimated using the EBICglasso estimator (*γ* = 0.50). Normalized centrality measures were calculated to compare the pre-COVID (PreCo), post-COVID/pre-invasion (PostCoPreW), and post-COVID/post-invasion-outbreak (PostCoPostW) samples. To evaluate the relative importance of variables within the network structures, betweenness, closeness, strength, and expected influence centrality indices were calculated. Edge-weight accuracy and centrality stability were assessed using 1,000 non-parametric and 1,000 case-dropping bootstrap iterations, respectively.

Statistical analyses were performed using IBM SPSS Statistics versions 29.0 and 31.0 and JASP (Version 0.98.1; JASP Team, 2026) for Windows. One-way ANOVAs, Games–Howell *post hoc* comparisons, Cohen’s *d* effect-size calculations, and network analyses were conducted using JASP.

## Results

### Sample characteristics

The demographic characteristics of the three independent samples are presented in **Table 1**. No significant differences were observed in sex distribution (*χ*^2^(2) = 3.193, *p* = 0.203) or age-group distribution (*χ*^2^(4) = 1.881, *p* = 0.758) across the three study periods. Significant differences were observed in occupational distribution (*χ*^2^(4) = 26.63, *p* < 0.001; Cramer’s *V* = 0.147) and loss experience (*χ*^2^(4) = 60.38, *p* < 0.001; Cramer’s *V* = 0.214), although both effect sizes were small.

**Table 1:** Demographic characteristics of the three independent samples and *χ*^2^ comparisons across the study periods.

Variable	Valid *N*	PreCo	PostCoPreW	PostCoPostW	χ^2^ (df)	p
		*N* = 249	*N* = 265	*N* = 148		
Sex	662				3.193 (2)	0.203
Male	100	39 (15.7)	33 (12.5)	28 (18.9)		
Female	562	210 (84.3)	232 (87.5)	120 (81.1)		
Age group	660				1.881 (4)	0.758
≤25 years	12	6 (2.4)	4 (1.5)	2 (1.4)		
26–54 years	532	204 (81.9)	209 (79.2)	119 (81.0)		
≥55 years	116	39 (15.7)	51 (19.3)	26 (17.7)		
Occupation	613				26.63 (4)	<0.001
Medical	76	9 (4.1)	48 (19.0)	19 (12.8)		
Helper	238	85 (38.6)	97 (38.3)	56 (40.0)		
Non-helper	299	126 (57.3)	108 (42.7)	65 (46.4)		
Loss	662				60.38 (4)	<0.001
No loss	251	137 (55.0)	73 (27.5)	41 (27.7)		
Death-related	285	92 (36.9)	127 (47.9)	66 (44.6)		
Other loss	126	20 (8.0)	65 (24.5)	41 (27.7)		

Values are presented as *n* (%). Percentages are calculated within each study period. Valid *N* refers to the number of participants with complete data for each variable. Comparisons were performed across the three independent samples.

*χ*^2^, Pearson chi-square test.

### Descriptive statistics

Descriptive statistics for all main study variables are presented in **Supplementary Table S1**.

### Associations

Overall correlation patterns remained largely stable across the three study periods. Only a limited number of statistically significant changes in correlation coefficients across the study periods were identified by Fisher’s *Z* analyses.

Associations between coping competencies and MIL were generally stronger following COVID-19 and remained stronger for most competencies after Russia’s full-scale invasion of Ukraine. However, social empathy and social mobilizing capacity no longer showed significant positive associations with MIL in the PostCoPostW sample, while social creating capacity, after showing a stronger association with MIL in the PostCoPreW sample, showed a weaker association in the PostCoPostW sample.

In contrast, correlations between coping competencies and overall FOD were generally weaker in the two post-COVID samples than in the PreCo sample. Detailed correlation coefficients are presented in **Tables 2**–**5**.

**Table 2:** Overall correlations between fear of death, coping subsystems, and meaning in life across crisis periods.

Variable	PreCo	PostCoPreW	PostCoPostW
MFODS Total × PISI Total	−0.296^**^	−0.242***	−0.223*
MFODS × MAS	−0.252**	−0.184**	−0.186*
MFODS × MCES	−0.181**	−0.166**	−0.066
MFODS × SRS	−0.354**	−0.296***	−0.372***
MLQ Total × PISI Total	0.334**	0.376***	0.443***
MLQ × MAS	0.351**	0.386***	0.485***
MLQ × MCES	0.346**	0.452***	0.368***
MLQ × SRS	0.156	0.084	0.271**
MFODS × MLQ Total	−0.045	−0.039	0.065
MFODS × MLQ-P	−0.245***	−0.167**	−0.155
MFODS × MLQ-S	0.159*	0.119	0.272**

Values are Pearson correlation coefficients (r). Correlations were calculated separately for each study period.

MFODS, Multidimensional Fear of Death Scale; MLQ, Meaning in Life Questionnaire; MLQ-P, presence of meaning; MLQ-S, search for meaning; PISI, Psychological Immune System Inventory; MAS, Monitoring Approaching Subsystem; MCES, Mobilizing, Creating and Executing Subsystem; SRS, Self-Regulating Subsystem.

*p < 0.05; **p < 0.01; ***p < 0.001.

**Table 3A:** Correlations between fear of death dimensions and meaning in life components across the three study periods.

Pearson’sr	PreCoN = 249			PostCoPreWN = 252			PostCoPostWN = 139		
Variables	MLQ-P	MLQ-S	MLQ	MLQ-P	MLQ-S	MLQ	MLQ-P	MLQ-S	MLQ
MFODS Total	−0.245**	0.159*	−0.045	−0.167**	0.119	−0.039	−0.155	0.272**	0.065
Fear of the dying process	−0.241***	0.113	−0.078	−0.173**	0.010	−0.058	−0.265**	0.157	−0.078
Fear of the dead	−0.159*	0.064	−0.060	−0.049	0.079	0.021	−0.034	0.141	0.065
Fear of being destroyed	0.012	0.062	0.056	0.055	−0.055	−0.002	0.135	**0**.**154**	**0**.183*
Fear for significant others	−0.203*	0.190**	0.008	**−0**.**057**	**0**.**028**	−0.021	−0.109	0.153	0.022
Fear of the unknown	−0.283***	0.009	−0.189**	−0.323***	0.060	−0.199**	−0.181*	0.115	−0.049
Fear of the conscious death	0.035	0.109	0.110	−0.040	0.096	0.038	0.034	**0**.325***	**0**.222*
Fear for the body after death	−0.119	0.096	−0.007	−0.055	0.032	−0.019	−0.075	0.123	0.026
Fear of premature death	−0.213***	0.137*	−0.040	−0.187**	0.217**	0.018	−0.233**	0.199*	−0.031

Values are Pearson correlation coefficients (*r*). Correlations were calculated separately for each study period. Bold values indicate correlations for which Fisher’s *Z* tests identified significant differences between study periods.

MFODS, Multidimensional Fear of Death Scale; MLQ, Meaning in Life Questionnaire; MLQ-P, presence of meaning; MLQ-S, search for meaning.

*p < 0.05; **p < 0.01; ***p < 0.001.

**Table 3B:** Correlations between overall FOD (MFODS Total) and PISI Total, its subsystems, and coping competencies across the three study periods.

Coping variables	PreCo	PostCoPreW	PostCoPostW
	MFODS	MFODS	MFODS
PISI	−0.296^**^	−0.242***	−0.223*
MAS	−0.252**	−0.184**	−0.186*
MCES	−0.181**	−0.166**	−0.066
SRS	−0.354**	−0.296***	−0.372***
Positive thinking	−0.276***	−0.168**	−0.261**
Sense of control	−0.052	0.041	−0.057
Sense of coherence	−0.241***	−0.242***	−0.206*
Self-respect	−0.123	−0.158*	−0.133
Sense of self-growth	−0.225***	−0.224***	−0.218*
Challenge and flexibility	−0.258***	−0.205**	−0.111
Social empathy	0.025	−0.025	0.034
Problem-solving capacity	−0.11	−0.096	−0.052
Sense of self-efficacy	−0.175**	−0.137*	−0.04
Social mobilizing capacity	−0.106	−0.112	−0.069
Social creating capacity	−0.172***	−0.154*	0.036
Synchronicity	−0.263***	−0.346**	−0.355***
Goal orientation	−0.126*	−0.019	−0.014
Impulse control	−0.212***	−0.010	−0.176*
Emotional control	−0.375***	−0.396***	−0.330***
Irritability control	−0.274***	−0.174**	−0.313***

Values are Pearson correlation coefficients (*r*). Correlations were calculated separately for each study period.

MFODS, Multidimensional Fear of Death Scale; PISI, Psychological Immune System Inventory; MAS, Monitoring Approaching Subsystem; MCES, Mobilizing, Creating and Executing Subsystem; SRS, Self-Regulating Subsystem.

*p < 0.05; **p < 0.01; ***p < 0.001.

**Table 4:** Correlations with statistically significant differences across the three study periods (Fisher’s *Z* transformation).

Variable pairs	PreCo	PostCoPreW	PostCoPostW
Fear of being destroyed × MLQ	0.056	0.002	0.183*
Fear of being destroyed × MLQ-S	0.062	−0.055	**0.154**
Fear for significant others × MLQ-P	−0.203**	−**0.057**	−0.109
Fear for significant others × MLQ-S	0.190**	**0.028**	0.153
Fear of conscious death × MLQ	0.011	0.038	**0**.222**
Fear of conscious death × MLQ-S	0.109	0.096	**0**.325***
MFODS × social creating capacity	−0.172**	−0.152*	**0.036**
MFODS Total × impulse control	−0.212***	−**0.011**	−0.176*
MLQ × sense of self-growth	0.324***	0.273***	**0**.503***
MLQ × social empathy	0.183**	0.288***	**0**.**097**
MLQ × social mobilizing capacity	0.196**	0.350***	**0.147**
MLQ × social creating capacity	0.333***	0.444***	0.275***
MLQ × impulse control	0.078	−0.040	0.236**
MLQ-P × social empathy	0.089	0.316***	**0**.**088**
MLQ-P × problem-solving capacity	0.202**	0.357***	0.199*
MLQ-P × social creating capacity	0.414***	0.444***	0.269***
MLQ-P × impulse control	0.139*	0.113	**0**.311***
MLQ-S × MLQ-P	−0.085	−0.078	0.231**
MLQ-S × sense of control	−0.039	0.029	0.204*
MLQ-S × sense of coherence	−0.112	−0.151*	**0.057**
MLQ-S × sense of self-growth	−0.084	−0.136*	**0.134**
MLQ-S × sense of self-efficacy	0.021	0.006	0.205*
MLQ-S × impulse control	−0.023	−0.165**	**0.043**

Values are Pearson correlation coefficients (*r*). Bold values indicate correlation coefficients involved in statistically significant differences identified by Fisher’s *Z* tests between study periods.

MLQ, Meaning in Life Questionnaire; MLQ-P, presence of meaning; MLQ-S, search for meaning.

*p < 0.05; **p < 0.01; ***p < 0.001.

Correlations between FOD dimensions and MIL showed heterogeneous patterns across the three study periods. MLQ-P generally exhibited weaker negative correlations with several FOD dimensions across the post-crisis samples, remaining significant for fewer FOD dimensions than before the pandemic.

Fisher’s *Z* analyses identified statistically significant differences in selected associations between MIL and FOD dimensions (**Table 4**). For overall MIL, significant differences were observed in the associations with fear of conscious death and fear of being destroyed. MLQ-P showed a significant change in its negative association with fear for significant others. Search for meaning exhibited significantly stronger positive associations with fear of conscious death and fear of being destroyed, whereas its association with fear for significant others became significantly weaker.

Fisher’s *Z* analyses also identified significant differences in associations between MIL and coping variables. Overall, MIL showed significant differences in its associations with sense of self-growth, social empathy, social mobilizing capacity, social creating capacity, and impulse control. MLQ-P exhibited significant differences in its associations with social empathy, sense of self-growth, problem-solving capacity, social creating capacity, and impulse control. MLQ-S showed significant differences in its associations with sense of control, sense of coherence, sense of self-growth, sense of self-efficacy, and impulse control (**Table 4**).

### Linear regression

Backward linear regression analyses were conducted to identify key predictors of FOD separately across the three study periods. As shown in **Table 5**, the pattern of significant predictors differed across the examined periods.

In the pre-COVID sample, emotional control was the strongest predictor of FOD (standardized *β* = −0.297), while MLQ-S also contributed independently to the model. In the post-COVID pre-war sample, emotional control remained the strongest predictor (standardized *β* = −0.378), together with impulse control and synchronicity. Following the outbreak of Russia’s full-scale invasion of Ukraine, the pattern of predictors differed from the previous periods. MLQ-S was again identified as a significant predictor (standardized *β* = 0.272), alongside positive thinking, social creating capacity, and irritability control.

### One-way analyses of variance

One-way ANOVAs were conducted to compare FOD, coping competencies, and MIL across the PreCo, PostCoPreW, and PostCoPostW samples. When overall group differences were significant, Games–Howell *post hoc* tests were performed because the assumption of homogeneity of variances was not consistently met. Effect sizes for pairwise comparisons were estimated using Cohen’s *d*.

Although mean total MFODS scores were higher in the PostCoPreW sample and lower in the PostCoPostW sample, differences in total MFODS scores across the three study periods did not reach statistical significance.

Among the FOD dimensions, significant between-group differences were observed for fear of being destroyed, fear for significant others, and fear of premature death. *Post hoc* comparisons indicated that these differences mainly involved the PostCoPreW sample, which showed higher scores than either the PreCo or the PostCoPostW sample, depending on the specific FOD dimension. The remaining FOD dimensions did not differ significantly across the three study periods.

Overall PISI competence (PISI Total) differed significantly across the study periods, with lower scores observed in both post-COVID samples than in the pre-COVID sample, whereas no significant difference was observed between the two post-COVID samples.

Within the MAS, sense of coherence, social empathy, sense of self-growth, and challenge and flexibility were significantly lower in the post-COVID samples than in the pre-pandemic sample, with no significant differences between the two post-COVID samples. Within the SRS, synchronicity and emotional control were significantly lower in the post-COVID samples. Although mean SRS scores were slightly higher in the PostCoPostW sample than in the PostCoPreW sample, this difference was not statistically significant.

MCES scores were significantly lower in the PostCoPreW sample. Although mean MCES scores increased slightly in the PostCoPostW sample, Games–Howell *post hoc* comparisons indicated no statistically significant differences either between the two post-COVID samples or between the PreCo and PostCoPostW samples.

No significant differences were observed for MLQ-P, MLQ-S, or MLQ Total across the three study periods.

**Table 6** presents the results of the one-way ANOVAs, Games–Howell *post hoc* comparisons, and Cohen’s *d* effect sizes.

**Table 5:** Backward linear regression models predicting FOD across the three study periods.

PreCo						PostCoPreW						PostCoPostW					
	UnStd. *β*		Std. *β*	*t*	*p*		UnStd. *β*		Std. *β*	*t*	*p*		UnStd. *β*		Std. *β*	*t*	*p*
	*β*	SE					*β*	SE					*β*	SE			
Positive thinking	−0.79	0.453	−0.12	−1.738	0.083	Synchronicity	−1	0.452	−0.168	−2.221	0.03	Positive thinking	−1.301	0.604	−0.223	−2.152	0.033
Emotional control	−1.83	0.427	−0.297	−4.282	<0.001	Impulse control	1.465	0.433	0.217	3.384	<0.001	Social creating capacity	1.396	0.572	0.215	2.442	0.016
MLQ-S	0.359	0.174	0.122	2.062	0.04	Emotional control	−2.3	0.483	−0.378	−4.752	<0.001	Synchronicity	−1.108	0.605	−0.184	−1.833	0.069
												Irritability control	−1.238	0.551	−0.197	−2.247	0.026
												MLQ-S	0.826	0.24	0.272	3.436	<0.001

Multicollinearity diagnostics indicated no substantial concerns across the regression models (VIFs ≤ 1.9; tolerance ≥ 0.54). Backward linear regression analyses were performed separately for each study period. Dependent variable: MFODS Total. Variables entered into the model: positive thinking, sense of coherence, self-respect, sense of self-growth, challenge and flexibility, sense of self-efficacy, social creating capacity, synchronicity, impulse control, emotional control, irritability control, and MLQ-S.

**Table 6:** One-way ANOVA, Games–Howell *post hoc* comparisons, and Cohen’s *d* effect sizes across the three study periods.

Variable	Mean (SD)			*F* (df1, df2)	Pairwise comparison Cohen’s *d*		
	1	2	3		1–2	2–3	1–3
MFODS	114 (22)	118 (22)	113 (22)	2.827 (2,638)	−0.189	0.197	0.007
Fear of the dying process	18.9 (5.7)	19.4 (5.4)	19 (5.3)	0.667 (2,656)	−0.097	0.079	−0.018
Fear of the dead	15.9 (5.0)	16.0 (5.2)	15.5 (4.7)	0.668 (2,657)	−0.023	0.117	0.093
Fear of being destroyed	9.2 (4.3)	10.3 (4.6)	9.5 (4.4)	3.789 (2,656)	−0.237*	0.177	−0.061
Fear for significant others	24.2 (4.1)	24.7 (4.5)	23.5 (4.9)	3.266 (2,656)*	–0.105	0.263*	0.158
Fear of the unknown	14.3 (3.6)	14.4 (3.6)	13.7 (3.7)	1.993 (2,651)	−0.028	0.199	0.171
Fear of conscious death	9.9 (3.5)	10.0 (3.9)	10.7 (4.2)	1.9 (2,651)	−0.048	−0.147	−0.204
Fear for the body after death	10.8 (4.2)	10.5 (4.3)	10.4 (4.3)	0.623 (2,657)	−0.098	0.028	−0.068
Fear of premature death	11.0 (4.6)	12.1 (4.7)	11.3 (4.5)	3.746 (2,655)**	−0.237*	0.168	−0.069
PISI	241 (33)	231 (35)	231 (37)	6.500 (2,632)**	0.292**	0.003	0.295*
MAS	109 (14)	105 (16)	104 (16)	6.354 (2,644)*	0.266**	0.055	0.320**
MCES	78 (12)	74 (14)	75 (14)	4.260 (2,645)*	0.251*	−0.054	0.196
SRS	55 (11)	52 (11)	53 (11)	4.444 (2,652)*	0.258*	−0.065	0.193
Positive thinking	15.4 (3.3)	14.9 (3.7)	14.9 (3.8)	1.632 (2,652)	0.141	0.011	0.152
Sense of control	14.0 (2.5)	13.8 (2.7)	13.9 (2.5)	0.661 (2,655)	0.101	−0.049	0.052
Sense of coherence	16.7 (2.9)	16.1 (3.1)	15.5 (3.8)	5.800 (2,653)**	0.171	0.181	0.352**
Self-respect	15.8 (3.2)	15.4 (3.4)	15.4 (3.7)	0.794 (2,656)	0.100	0.103	0.003
Sense of self-growth	16.4 (3.1)	15.6 (3.2)	15.5 (3.6)	4.743 (2,655)**	0.232*	0.038	0.270*
Challenge and flexibility	15.2 (3.5)	13.9 (3.9)	14.4 (3.9)	7.459 (2,655)***	0.340***	−0.130	0.210
Social empathy	15.7 (3.1)	15.0 (3.4)	14.8 (3.2)	5.027 (2,653)**	0.233*	0.053	0.286*
Problem-solving capacity	16.1 (3.0)	15.1 (3.5)	15.6 (3.3)	5.932 (2,655)**	0.303**	0.130	−0.173
Sense of self-efficacy	15.6 (3.0)	15.2 (3.0)	15.3 (3.4)	1.167 (2,654)	0.132	0.100	−0.032
Social mobilizing capacity	15.0 (3.4)	14.5 (3.7)	14.5 (3.7)	1.542 (2,649)	0.135	0.152	0.018
Social creating capacity	15.2 (3.1)	14.0 (3.7)	14.1 (3.5)	8.132 (2,654)***	0.337***	−0.041	0.297*
Synchronicity	14.2 (3.7)	13.1 (3.6)	13.2 (3.8)	6.875 (2,656)**	0.299**	−0.003	0.296*
Goal orientation	15.4 (3.1)	15.3 (3.1)	15.0 (3.1)	0.688 (2,656)	0.022	0.119	0.097
Impulse control	14.8 (2.9)	14.5 (3.2)	14.9 (3.1)	1.089 (2,654)	0.110	−0.019	−0.128
Emotional control	12.5 (3.6)	11.6 (3.6)	12.0 (3.7)	4.146 (2,655)*	0.254*	−0.107	0.147
Irritability control	13.1 (3.7)	12.5 (3.6)	12.6 (3.6)	1.765 (2,655)	0.159	0.132	−0.027
MLQ Total	50.5 (9.5)	51.2 (9.4)	50.9 (11.9)	0.277 (2,656)	−0.065	0.024	−0.042
MLQ-P	26.6 (6.5)	26.0 (7.0)	26.6 (7.9)	1.073 (2,657)	0.089	0.057	0.145
MLQ-S	23.9 (7.4)	25.2 (6.9)	25.3 (7.3)	2.794 (2,656)	−0.183	−0.018	−0.201

1, PreCo; 2, PostCoPreW; 3, PostCoPostW.

MFODS, Multidimensional Fear of Death Scale; MLQ, Meaning in Life Questionnaire; MLQ-P, presence of meaning; MLQ-S, search for meaning; PISI, Psychological Immune System Inventory; MAS, Monitoring Approaching Subsystem; MCES, Mobilizing, Creating and Executing Subsystem; SRS, Self-Regulating Subsystem.

*p < 0.05; **p < 0.01; ***p < 0.001.

### Network analysis

To examine patterns of associations among FOD dimensions, MIL, and coping competencies across the three study periods, networks were estimated separately for the PreCo, PostCoPreW, and PostCoPostW samples.

In **Figures 1A–C**, lines between nodes represent associations between variables, with thicker lines indicating stronger relationships. Blue lines indicate positive associations, whereas red lines indicate negative associations. Network centrality measures included betweenness, closeness, strength, and expected influence. **Figure 1A** presents the PreCo network, **Figure 1B** the PostCoPreW network, and **Figure 1C** the PostCoPostW network. Orange nodes represent PISI coping competencies, blue nodes represent MFODS dimensions, and green nodes represent MLQ-S and MLQ-P. The standardized centrality indices are shown in **Figure 2**

**Figure 1:**
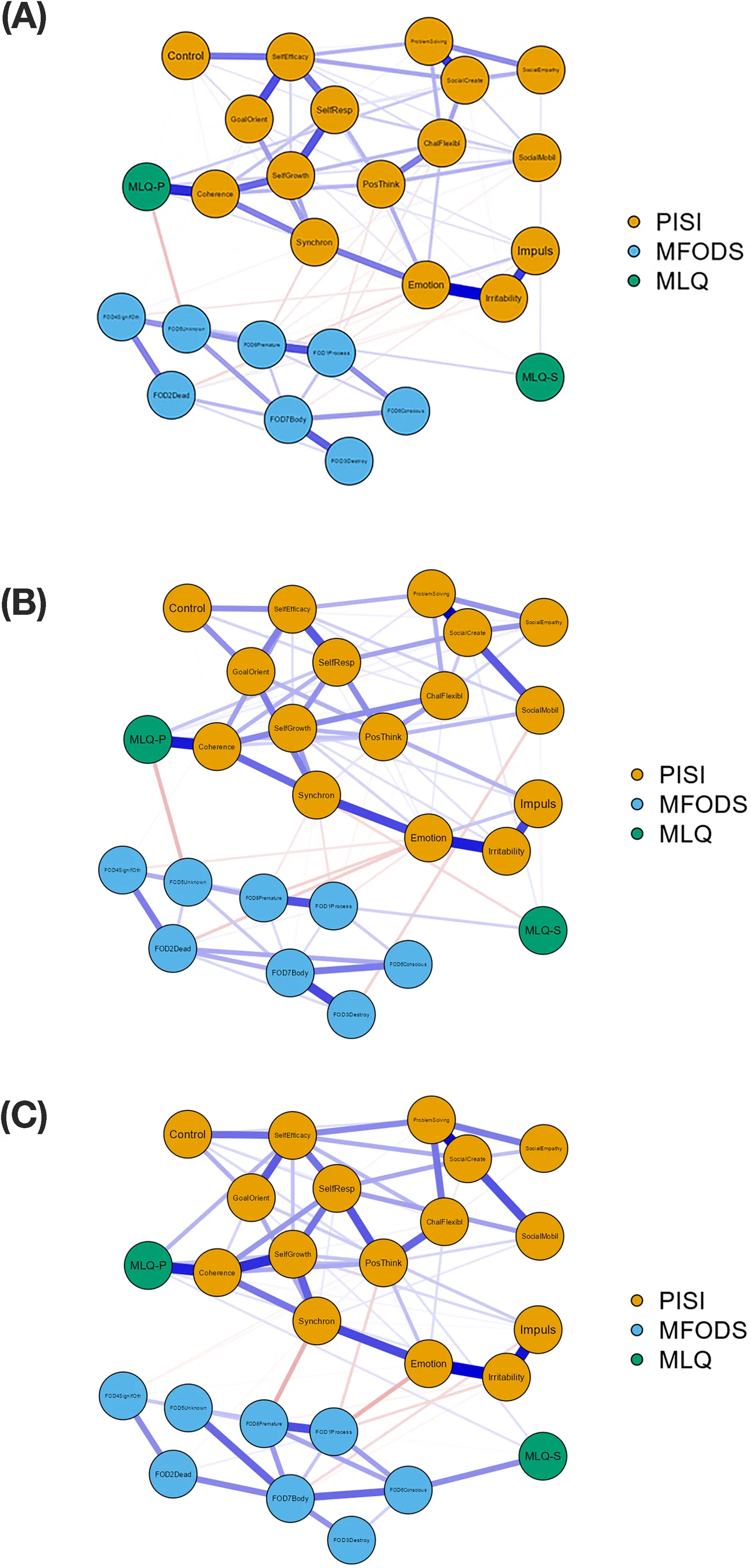
Network analysis of FOD, MIL, and coping competencies across the three study periods. Orange nodes represent PISI coping competencies, blue nodes represent MFODS dimensions, and green nodes represent the Meaning in Life Questionnaire (MLQ): MLQ-P, presence of meaning; MLQ-S, search for meaning. Blue edges indicate positive associations, and red edges indicate negative associations. Edge thickness reflects the strength of the association.

Across the networks, coping competencies were predominantly positively connected with MIL variables. MFODS dimensions showed predominantly negative connections with coping competencies and MLQ-P, whereas several positive connections were observed with MLQ-S.

**Figure 1C** indicates a more integrated pattern involving both MIL dimensions following the outbreak of Russia’s full-scale invasion of Ukraine. In particular, MLQ-S appeared more integrated within the network, while the association between MLQ-S and MLQ-P became more pronounced in the PostCoPostW network.

Self-respect, sense of self-efficacy, and sense of coherence showed relatively high expected influence values across the networks, while MLQ-P remained closely connected to sense of coherence. Synchronicity showed higher betweenness centrality in the PostCoPreW and PostCoPostW networks than in the PreCo network, with the highest value observed in the PostCoPreW network. In addition, different patterns of associations between MFODS dimensions and coping competencies were observed across the three networks, including differences in the strength and direction of several connections.

Components of the SRS remained connected to FOD dimensions across the examined three study periods. In the PostCoPostW network, the pattern of connections involving the MLQ dimensions and components of the SRS differed from that observed in the earlier networks (**Figure 1C**).

**Figure 2:**
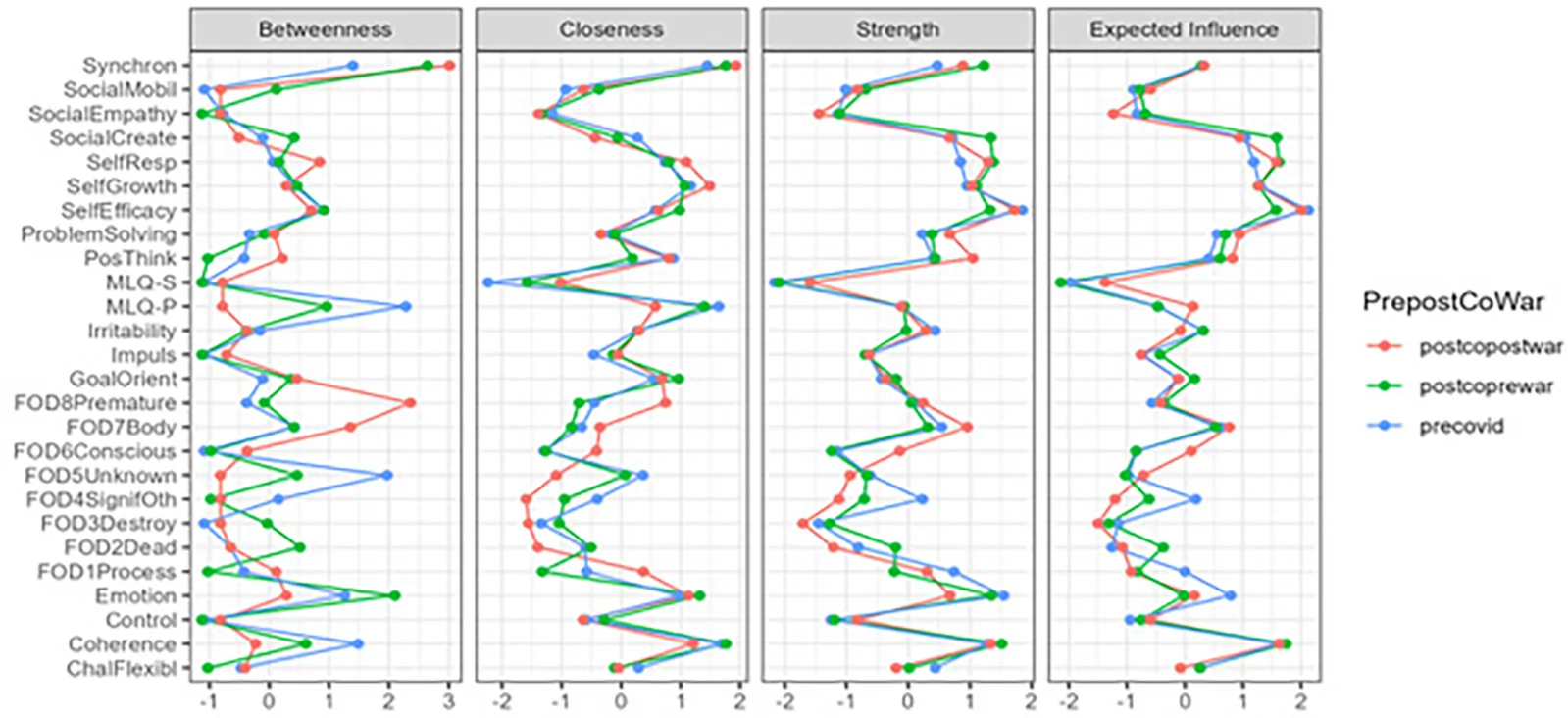
Standardized centrality indices across the three study periods. Standardized centrality indices (betweenness, closeness, strength, and expected influence) for the network models across the three study periods: PreCo, PostCoPreW, and PostCoPostW. Higher standardized values indicate greater centrality of the corresponding node within the network. Abbreviations: MLQP, presence of meaning; MLQS, search for meaning; Synchron, synchronicity; SocialMobil, social mobilizing capacity; SocialEmpathy, social empathy; SocialCreat, social creating capacity; SelfResp, self-respect; SelfGrow, sense of self-growth; SelfEfficacy, sense of self-efficacy; ProblemSolving, problem-solving capacity; PosThink, Positive Thinking; Irritability, irritability control; Impuls, impulse control; GoalOrient, goal orientation; Emotion, emotional control; Control, sense of control; Coherence, sense of coherence; ChalFlexibil, challenge and flexibility; FOD1Process, fear of the dying process; FOD2, fear of the dead; FOD3, fear of being destroyed; FOD4, fear for significant others; FOD5, fear of the unknown; FOD6, fear of conscious death; FOD7, fear of the body after death; FOD8, fear of premature death (see specific dimensions on the *y*-axis).

At the subsystem level, MAS showed relatively high strength and expected influence values across all three networks. In contrast, differences in the role of MIL were primarily reflected in the reorganization of network connections rather than marked increases in centrality.

To further examine the organization of these relationships, **Supplementary Figures S1A–C** present an additional NA at the subsystem level, including the coping subsystems (MAS, MCES, and SRS), MFODS Total, and the two MIL dimensions (MLQ-P and MLQ-S).

Across all three study periods, MAS showed strong connections within the subsystem-level networks, including positive connections with the MIL dimensions. The association between MLQ-P and MLQ-S was stronger in the PostCoPostW network, accompanied by a different pattern of positive connections between the MIL dimensions and the coping subsystems. In contrast, MFODS Total remained negatively connected to the coping subsystems and showed only limited direct associations with the MIL dimensions.

## Discussion

The present study examined the associations between coping, MIL, and FOD during the early phase of Russia’s full-scale invasion of Ukraine in independent Hungarian samples from a population living in geographical proximity to the armed conflict. The study included medical professionals, other helping professionals, and non-helping professionals and examined differences in FOD, coping, and MIL, as well as their associations across study periods. In addition to identifying between-sample differences, the findings suggest a more integrative role of MIL within the network structure linking FOD and coping resources in the PostCoPostW sample. To date, relatively little is known about how FOD, coping competencies, and MIL are associated during the early phase of Russia’s full-scale invasion of Ukraine, particularly in the context of concurrent pandemic- and war-related existential threats. To our knowledge, no previous study examined these constructs simultaneously in independent samples collected before the COVID-19 pandemic, following a major COVID-19 mortality wave, and during the early phase of Russia’s full-scale invasion of Ukraine. Accordingly, the present study investigated differences in FOD, MIL, and coping competences, as well as their associations, across three independent samples collected during distinct societal periods: before the COVID-19 pandemic, following a major COVID-19 mortality wave, and during the early phase of Russia’s full-scale invasion of Ukraine.

Pavlova et al. (12) reported associations between anxiety, stress, depression, and life satisfaction among university students in Ukraine during the COVID-19 pandemic and Russia’s full-scale invasion of Ukraine. Their findings suggested that prevention programs should primarily target anxiety as a key stress-related factor, which may indirectly contribute to improved life satisfaction through reduced stress. These findings are consistent with our results, which showed that coping competencies remained closely associated with FOD across all three study periods despite differences in the strength of selected associations, and with previous studies reporting positive associations between stress and fear of death (59, 61). Previous research has also demonstrated that MIL is positively associated with coping competencies, whereas MLQ-P is negatively and MLQ-S is positively associated with FOD (62), consistent with the overall pattern observed in the present study. Furthermore, studies conducted during the H1N1 pandemic showed that greater intolerance of uncertainty was associated with higher levels of anxiety and a tendency to use emotion-focused coping strategies (26).

The overall results suggest less favorable coping profiles in the PostCoPreW and PostCoPostW samples compared with the PreCo sample. Synchronicity emerged as a relevant coping competence, reflecting the ability to adapt to changing circumstances and challenging situations. A similar pattern was observed for MLQ-S, suggesting that active meaning seeking may represent an adaptive response when individuals are confronted with uncertainty and existential threats. Although the overall correlations were modest, both the network analysis and Fisher’s *Z* transformations indicated closer associations between MLQ-S and selected FOD dimensions in the PostCoPostW sample, consistent with theoretical considerations (45, 62). These findings may reflect greater engagement in meaning-seeking processes under conditions of heightened uncertainty. Although MLQ-S scores were descriptively higher in the PostCoPostW sample than in the PostCoPreW sample, this difference was not statistically significant. Its increased relevance across the association-based analyses nevertheless suggests that meaning seeking may have been particularly salient during the early phase of Russia’s full-scale invasion of Ukraine. Notably, Fisher’s *Z* analyses indicated stronger associations between MLQ-S and fear of conscious death and fear of being destroyed in the PostCoPostW sample, further supporting the relevance of meaning-related processes under existential threat.

Different patterns of associations were observed between FOD dimensions and the MLQ factors across the three study periods. International research has consistently identified fear of the dying process, fear of the dead, fear for significant others (which includes concerns related to one’s own death), and fear of premature death as the dimensions associated with the highest levels of fear (55, 61). In the PreCo network, MLQ-P showed a negative connection with fear of the unknown, which was no longer apparent in the PostCoPostW network.

MLQ-P showed predominantly negative associations with several FOD dimensions, particularly in the PreCo sample. However, these associations were generally less pronounced in the post-crisis samples, with Fisher’s *Z* analyses identifying a significant difference only for fear for significant others. In contrast, MLQ-S showed a different pattern of associations with FOD dimensions across the study periods. Fisher’s *Z* analyses indicated stronger associations with fear of conscious death and fear of being destroyed, whereas the association with fear for significant others became weaker. In addition, Fisher’s *Z* analyses demonstrated that the association between sense of self-growth and MIL was significantly stronger in the PostCoPostW sample than in the earlier samples. This finding suggests that experiences related to personal growth were more closely linked to the experience of a meaningful life despite the overall decline observed in several coping competencies.

Before the pandemic, fear for significant others was positively associated with MLQ-S. However, this association was significantly weaker in the subsequent study periods, whereas stronger associations were observed between MLQ-S and fear of conscious death and fear of being destroyed. Although fear for significant others encompasses concerns related to one’s own death, the stronger post-crisis associations between MLQ-S and fear of conscious death and fear of being destroyed are likewise consistent with previous MIL and TMT research, suggesting that heightened mortality awareness may be associated with an increased tendency to search for meaning (2, 48). Experiences of loss and bereavement may also contribute to FOD related to significant others. The death of a loved one may challenge previously held assumptions regarding coherence, meaning, and predictability in life. Similar meaning-seeking tendencies have been described in bereavement and grief-related literature (63).

Compared with the PreCo sample, the PostCoPreW sample showed higher levels of fear of premature death, while MLQ-S emerged as relevant across several association-based analyses. In the PostCoPostW sample, MLQ-S remained consistently relevant across the various statistical analyses. At the same time, lower levels of fear for significant others, together with differences in specific coping competencies and the different pattern of associations involving MLQ-S, may reflect a shift in death-related concerns, with meaning-related coping processes becoming more prominent during the early phase of Russia’s full-scale invasion of Ukraine. The combined context of the COVID-19 pandemic and the war may have increased coping demands, potentially contributing to the greater relevance of meaning-related coping processes within the less favorable coping profiles observed in the later study periods.

During the early phase of Russia’s full-scale invasion of Ukraine, irritability control emerged as a relevant predictor of FOD, whereas emotional control was a prominent predictor in the PreCo and particularly in the PostCoPreW sample. This pattern may reflect greater strain on emotional regulation under prolonged population-level stressors. Such an interpretation is consistent with burnout-related processes, in which irritability and heightened emotional reactivity are common symptoms and may involve stronger responses to stressors that might be perceived as manageable under psychologically stable conditions. Recent studies suggest that burnout-related phenomena are not exclusively associated with occupational stressors (64). It is also possible that pandemic-related lockdowns, work-related uncertainties, and prolonged ambiguity had already contributed to adverse psychological effects before the outbreak of the invasion, potentially influencing the patterns observed in the PostCoPostW sample.

The descriptively lower FOD levels and higher MLQ-S observed in the PostCoPostW sample, despite concurrent societal stressors, together with the less favorable coping profile observed in the later study periods, may be interpreted within the framework of TMT, particularly its concept of distal defense mechanisms. The relatively prominent network positions of self-respect and self-efficacy, together with the stronger association between sense of self-growth and MIL identified by Fisher’s *Z* analyses, may also be consistent with this interpretation. From this perspective, the descriptively lower FOD levels observed alongside changes in coping and meaning-related variables may indicate a shift from consciously experienced death-related concerns toward more indirect existential coping processes. According to TMT, when mortality-related concerns are managed primarily through defensive processes rather than conscious reflection, less adaptive individual and social responses may occur. The relevance of MLQ-S across the correlation, Fisher’s *Z*, regression, and NA is also consistent with this interpretation, although these findings should be understood as indirect evidence rather than a direct assessment of distal defense processes. NA further suggested that differences in the role of MIL were primarily reflected in the pattern of network connections, while centrality differences were interpreted cautiously due to their limited stability. In particular, the stronger association between MLQ-P and MLQ-S in the PostCoPostW network, together with FOD variables and coping resources, may indicate a closer integration of meaning-related processes under prolonged existential threat.

From a public mental health perspective, descriptively lower FOD in the presence of reduced coping resources may indicate potential vulnerability to adverse psychological outcomes during prolonged societal crises. This interpretation may be particularly relevant in societies with collective historical memories of geopolitical threats. According to TMT, proximal defense mechanisms are activated immediately in response to mortality-related threats (20). Beyond the immediate defensive reactions, effective emotional regulation may facilitate more adaptive coping. NA further indicated that components of the SRS, particularly synchronicity, remained connected to FOD across the study periods.

Conversely, theoretical accounts suggest that conscious reflection on mortality may facilitate adaptive coping by promoting reflective processing rather than automatic defensive responses (20). However, the form in which mortality awareness is experienced appears to be important. Grant and Wade-Benzoni’s dual-process model of death awareness distinguishes between “hot” and “cool” processing of mortality (2). “Hot” processing refers to immediate, emotion-driven reactions associated with death anxiety, whereas “cool” processing involves cognitive reflection on mortality, MIL, and personal values, potentially facilitating more prosocial responses (2). Weierstall-Pust et al. (14) suggested that, in the context of the Ukraine crisis, compassion may serve as an internal locus of control and stress-regulating resource, promoting helping behavior as a more reflective and prosocial response to existential threat. In this context, volunteering and helping behaviors may provide opportunities to channel mortality-related concerns into meaningful and socially constructive actions, consistent with the relevance of meaning-related processes observed across several analyses in the present study.

Previous research demonstrated positive associations between psychological immune competence, MIL, and MLQ-P, with the strongest relationship consistently observed between sense of coherence and MLQ-P (62). This association remained apparent across the PostCoPreW and PostCoPostW networks despite lower levels of sense of coherence in the later study periods. Sense of coherence is closely related to the construct of MIL as it reflects the perception of life as comprehensible, manageable, and meaningful (50). It also encompasses continuity and coherence across past, present, and future experiences. Large-scale societal stressors such as the COVID-19 pandemic and Russia’s full-scale invasion of Ukraine may challenge this sense of continuity and coherence. According to Antonovsky, sense of coherence comprises three interrelated components: comprehensibility, manageability, and meaningfulness. Together with purpose and significance, these dimensions contribute to adaptive coping (50). The pandemic and Russia’s full-scale invasion of Ukraine represent societal stressors over which individuals have limited control, potentially affecting the manageability component of sense of coherence. The invasion may also challenge comprehensibility and meaningfulness, potentially increasing the relevance of meaning-making processes. Similar reductions in perceived control and sense of coherence in the context of the war have been reported by Weierstall-Pust et al. (14). Although sense of coherence was lower in the PostCoPostW sample, the association between MLQ-P and MLQ-S was stronger in the PostCoPostW network, suggesting that these two dimensions may have been more closely interconnected under conditions of ongoing existential uncertainty. Consistent with this interpretation, the network shown in **Figure 1C** illustrates a more integrated pattern of connections involving the two MLQ dimensions, coping competencies, and FOD variables.

It is important to note that the present findings reflect the immediate and short-term period following Russia’s full-scale invasion of Ukraine. To our knowledge, relatively few studies have examined this early phase, and most available data were collected at least several weeks after the outbreak of the war, primarily in populations directly affected by the conflict. Xu et al. (34) investigated the psychological consequences during this early stage and observed high levels of perceived stress, depressive symptoms, and anxiety, highlighting the need for timely psychological interventions (34). In contrast, Pavlova et al. (12) reported more adaptive coping strategies among Ukrainian university students 6 months after the outbreak of the invasion, which they interpret as indicating psychological resilience over time.

Interestingly, Weierstall-Pust et al. (14), who analyzed the impact of stress related to the COVID-19 pandemic, climate change, and the Ukraine crisis in Germany 2 months after the outbreak of the invasion, reported that COVID-19 remained the strongest predictor of stress symptoms and health status (14).

In contrast, our findings showed lower levels of several coping-related resources in the PostCoPreW and PostCoPostW samples than in the PreCo sample, including sense of self-growth and sense of coherence. These differences were accompanied by distinct patterns in meaning-related processes. At the same time, the relevance of MLQ-S across the correlation, Fisher’s *Z*, regression, and NA, together with differences in self-related coping resources, may be interpreted within the framework of TMT as being consistent with meaning-related distal defense processes under conditions of existential threat. Lass-Hennemann et al. (4) reported increased anxiety levels associated with war-related threats among adolescents.

Psychological balance may become disrupted when individuals experience a loss of control over important life events. From a mental health perspective, maintaining a coherent and meaningful worldview may therefore be particularly important when individuals face changing challenges and external stressors. Such meaning systems integrate beliefs about control, coherence, values, and life goals, providing a framework through which individuals interpret experiences and maintain continuity between past, present, and future (65). These meaning structures may become particularly important when external circumstances are unpredictable and previously stable assumptions about life are challenged.

In the context of recent and ongoing global crises, people’s capacity for meaning-making and meaning reconstruction may play an important role in maintaining psychological balance and resilience (43, 44, 66, 67). This process may be supported by adaptive personality characteristics and psychological immune competencies that facilitate coping with mortality-related concerns in everyday life, consistent with the pattern of closer integration between meaning-related processes and coping resources observed in the present study.

The findings suggest that the COVID-19 pandemic and Russia’s full-scale invasion of Ukraine were associated with distinct patterns of FOD, MIL, and coping competencies and their relationships across the three study periods. While overall FOD showed higher descriptive levels in the PostCoPreW sample and lower descriptive levels in the PostCoPostW sample, significant between-sample differences were observed in several coping competencies, including sense of coherence, sense of self-growth, social empathy, challenge and flexibility, problem-solving capacity, synchronicity, and emotional control. Overall, these findings indicated less favorable coping profiles in the later study periods, although the specific pairwise differences varied across competencies.

At the same time, MLQ-S emerged as a relevant variable across the correlation, Fisher’s *Z*, regression, and NA, particularly in the PostCoPostW sample. The pattern of connections among MLQ-P, MLQ-S, and coping competencies, together with the stronger association between MIL and sense of self-growth, in the PostCoPostW sample, suggests a closer integration of meaning-related processes and coping resources. The relevance of MLQ-S across several analyses, alongside lower levels of sense of coherence, may further suggest that meaning-making processes represent an important adaptive resource during prolonged societal crises.

Given the repeated cross-sectional design with independent samples, the findings should be interpreted as between-sample differences observed across three study periods rather than within-person longitudinal changes or causal effects.

### Limitations and strengths

The study relied on convenience sampling through selected online channels; therefore, the findings cannot be considered representative of the Hungarian population. Although the samples were comparable regarding sex and age, small but statistically significant differences were observed in occupational composition and loss experience. Women were also overrepresented in all three samples, partly reflecting their predominance in helping professions. In addition, some demographic variables could not be compared consistently across the three study periods: regional information was available only for the PostCoPostW sample, while religiosity was assessed using different instruments. These sample characteristics should be considered when interpreting the findings and their generalizability.

Because three independent samples were examined rather than the same individuals over time, within-person changes and causal relationships cannot be inferred. Moreover, data were collected during three distinct historical periods characterized by different societal circumstances; therefore, unmeasured contextual factors unrelated to the COVID-19 pandemic or Russia’s full-scale invasion of Ukraine may also have contributed to the observed between-sample differences. The psychological mechanisms underlying the observed associations could not be directly examined.

Individual exposure to war-related events and media consumption related to the pandemic or the invasion were not assessed, limiting the examination of variability in participants’ exposure and perceptions. Furthermore, the third sample was collected during the early phase following the outbreak of Russia’s full-scale invasion of Ukraine. Consequently, these findings reflect immediate or short-term patterns and should not be interpreted as representing longer-term adaptation.

Given the large number of statistical comparisons conducted, the probability of type I error may be inflated. As no overall correction for multiple testing was applied across the exploratory association analyses, the reported findings should be interpreted with appropriate caution and warrant replication in future studies.

The NA also has methodological limitations. Non-parametric bootstrap analyses indicated variable precision of individual edge-weight estimates, particularly for weaker edges, while case-dropping bootstrap analyses showed limited stability of centrality estimates across the three networks. Therefore, individual edge weights and differences in relative node centrality should be interpreted cautiously, and the network findings should be considered primarily as exploratory patterns of associations rather than evidence of stable differences in network structure across the study periods.

Some MFODS and PISI subscales showed relatively low internal consistency, which may have attenuated associations and reduced the precision of the corresponding estimates. Analyses involving subscales with lower reliability should therefore be interpreted with particular caution. Finally, all variables were assessed using self-report questionnaires and may have been influenced by recall bias, response styles, social desirability, and limited introspective accuracy.

Despite these limitations, the study has several important strengths, including the use of validated psychological instruments and the comparison of three independent samples collected during three key societal periods, including the immediate phase following Russia’s full-scale invasion of Ukraine. A further strength is the convergence of findings across multiple complementary statistical approaches. Although individual associations varied across analyses and study periods, the correlation analyses, Fisher’s *Z* transformations, regression models, ANOVAs, and network analyses showed broadly consistent patterns and tendencies regarding the relationships among FOD, MIL, and coping resources. This convergence across analytical approaches strengthens confidence in the overall pattern of the findings, while individual estimates should still be interpreted with the methodological cautions described above.

### Highlight of the research

The COVID-19 pandemic and the outbreak of Russia’s full-scale invasion of Ukraine were associated with distinct differences in FOD, MIL, and coping competencies across the three study periods. Compared with the PostCoPreW sample, descriptively lower levels of FOD were observed in the PostCoPostW sample, while several coping resources were lower in both post-crisis samples than in the PreCo sample. MLQ-S also emerged as a relevant variable across multiple analyses, particularly in the PostCoPostW sample. These patterns may be interpreted within the framework of TMT as being consistent with meaning-related distal defense processes during prolonged societal crises, although these mechanisms were not directly assessed in the present study.

## Data Availability

The raw data supporting the conclusions of this article will be made available by the authors, without undue reservation.
