# Supplementary material for: Facing the unforeseen: coping, meaning in life, fear of death, and their associations across the COVID-19 pandemic and the early phase of Russia’s full-scale invasion of Ukraine—a repeated cross-sectional study in independent Hungarian samples
Source: Front Psychiatry. 2026 Oct 28;17:1916005. doi: 10.3389/fpsyt.2026.1916005 (PMC13753971; doi:10.3389/fpsyt.2026.1916005)
Supplement: Supplementary file 1 [file Table1.docx]

Supplementary Table S1.

*Descriptive statistics for all study variables across the three independent samples.*

|  | **PreCo** | | | | | | **PostCoPreW** | | | | | **PostCoPostW** | | | | |
| --- | --- | --- | --- | --- | --- | --- | --- | --- | --- | --- | --- | --- | --- | --- | --- | --- |
|  | **Valid N** | **Min** | **Max** | **Mean** | **SD** | **Valid N** | | **Min** | **Max** | **Mean** | **SD** | **Valid N** | **Min** | **Max** | **Mean** | **SD** |
| MFODS Total | 249 | 69 | 176 | 113.57 | 21.898 | 253 | | 66 | 176 | 117.7 | 21.516 | 139 | 55 | 174 | 113.41 | 22.196 |
| PISI Total | 249 | 146 | 304 | 241.11 | 32.808 | 252 | | 129 | 297 | 230.97 | 35.452 | 134 | 120 | 293 | 230.88 | 36.688 |
| Fear of the Dying Process | 249 | 6 | 30 | 18.9 | 5.745 | 264 | | 7 | 30 | 19.44 | 5.358 | 146 | 7 | 30 | 19 | 5.314 |
| Fear of the Dead | 249 | 6 | 30 | 15.95 | 5.009 | 264 | | 6 | 29 | 16.07 | 5.171 | 147 | 6 | 28 | 15.48 | 4.753 |
| Fear of Being Destroyed | 249 | 4 | 20 | 9.21 | 4.31 | 261 | | 4 | 20 | 10.27 | 4.615 | 147 | 4 | 20 | 9.48 | 4.372 |
| Fear for Significant Others | 249 | 11 | 30 | 24.21 | 4.144 | 263 | | 6 | 30 | 24.68 | 4.494 | 147 | 8 | 30 | 23.51 | 4.866 |
| Fear of the Unknown | 249 | 5 | 25 | 14.3 | 3.637 | 260 | | 9 | 25 | 14.4 | 3.565 | 145 | 8 | 25 | 13.68 | 3.672 |
| Fear of Conscious Death | 249 | 5 | 23 | 9.92 | 3.543 | 264 | | 5 | 25 | 10.09 | 3.968 | 147 | 5 | 25 | 10.69 | 4.162 |
| Fear for the Body after Death | 249 | 6 | 24 | 10.08 | 4.229 | 263 | | 6 | 28 | 10.49 | 4.263 | 148 | 6 | 27 | 10.37 | 4.506 |
| Fear of Premature Death | 249 | 4 | 20 | 11 | 4.572 | 262 | | 4 | 20 | 12.09 | 4.74 | 147 | 4 | 20 | 11.31 | 4.493 |
| Positive Thinking | 249 | 6 | 20 | 15.4 | 3.333 | 261 | | 5 | 20 | 14.89 | 3.662 | 145 | 5 | 20 | 14.86 | 3.786 |
| Sense of Control | 249 | 6 | 20 | 14.08 | 2.467 | 264 | | 8 | 20 | 13.81 | 2.699 | 145 | 7 | 19 | 13.94 | 2.546 |
| Sense of Coherence | 249 | 7 | 20 | 16.67 | 2.85 | 263 | | 6 | 20 | 16.13 | 3.125 | 144 | 5 | 20 | 15.55 | 3.788 |
| Self-Respect | 249 | 6 | 20 | 15.79 | 3.214 | 264 | | 5 | 20 | 15.45 | 3.387 | 146 | 6 | 20 | 15.44 | 3.682 |
| Sense of Self Growth | 249 | 6 | 20 | 16.38 | 3.075 | 264 | | 5 | 20 | 15.62 | 3.235 | 145 | 5 | 20 | 15.5 | 3.586 |
| Challenge and Flexibility | 249 | 5 | 20 | 15.18 | 3.452 | 263 | | 5 | 20 | 13.91 | 3.869 | 146 | 5 | 20 | 14.4 | 3.933 |
| Social Empathy | 249 | 5 | 20 | 15.75 | 3.105 | 262 | | 5 | 20 | 14.99 | 3.38 | 145 | 6 | 20 | 14.82 | 3.21 |
| Problem-Solving Capacity | 249 | 6 | 20 | 16.07 | 3.027 | 264 | | 5 | 20 | 15.07 | 3.512 | 145 | 7 | 20 | 15.64 | 3.284 |
| Sense of Self -Efficacy | 249 | 7 | 20 | 15.56 | 2.982 | 264 | | 5 | 20 | 15.15 | 3.043 | 144 | 5 | 20 | 15.25 | 3.354 |
| Social Mobilizing Capacity | 249 | 5 | 20 | 14.99 | 3.421 | 260 | | 5 | 20 | 14.51 | 3.668 | 143 | 5 | 20 | 14.45 | 3.65 |
| Social Creating Capacity | 249 | 7 | 20 | 15.15 | 3.142 | 264 | | 5 | 20 | 13.99 | 3.688 | 144 | 5 | 20 | 14.13 | 3.469 |
| Synchronicity | 249 | 5 | 20 | 14.25 | 3.692 | 264 | | 5 | 20 | 13.15 | 3.623 | 146 | 5 | 20 | 13.16 | 3.783 |
| Goal Orientation | 249 | 7 | 20 | 15.39 | 3.074 | 264 | | 5 | 20 | 15.33 | 3.072 | 146 | 7 | 20 | 15.03 | 3.109 |
| Impulse Control | 249 | 7 | 20 | 14.81 | 2.857 | 264 | | 5 | 20 | 14.48 | 3.179 | 144 | 6 | 20 | 14.87 | 3.103 |
| Emotional Control | 249 | 5 | 20 | 12.54 | 3.558 | 263 | | 5 | 20 | 11.63 | 3.554 | 146 | 5 | 20 | 12.01 | 3.684 |
| Irritability Control | 249 | 5 | 20 | 13.11 | 3.657 | 263 | | 5 | 20 | 12.53 | 3.62 | 146 | 5 | 20 | 12.63 | 3.551 |
| MAS | 249 | 65 | 136 | 108.84 | 14.418 | 257 | | 47 | 139 | 104.72 | 16.035 | 141 | 55 | 135 | 103.87 | 16.439 |
| MCES | 249 | 42 | 99 | 77.56 | 11.925 | 260 | | 29 | 100 | 74.25 | 13.726 | 139 | 36 | 99 | 74.97 | 14.183 |
| SRS | 249 | 28 | 79 | 54.71 | 11.066 | 262 | | 24 | 74 | 51.85 | 11.173 | 144 | 23 | 76 | 52.58 | 11.063 |
| MLQ-P | 249 | 5 | 35 | 26.63 | 6.536 | 263 | | 5 | 35 | 26.01 | 7.001 | 148 | 5 | 35 | 25.61 | 7.864 |
| MLQ-S | 249 | 5 | 35 | 23.9 | 7.419 | 263 | | 5 | 35 | 25.21 | 6.88 | 148 | 5 | 35 | 25.34 | 7.256 |
| MLQ Total | 249 | 13 | 70 | 50.53 | 9.463 | 262 | | 16 | 70 | 51.18 | 9.437 | 148 | 17 | 70 | 50.95 | 11.868 |

*Note. Valid N varies across variables because cases with missing item responses were excluded only from analyses involving the respective variables. PreCo = pre-COVID sample; PostCoPreW = post-COVID pre-war sample; PostCoPostW = post-COVID post-war sample.*
